# Bluetooth Based Chaos Synchronization Using Particle Swarm Optimization and Its Applications to Image Encryption

**DOI:** 10.3390/s120607468

**Published:** 2012-06-01

**Authors:** Her-Terng Yau, Tzu-Hsiang Hung, Chia-Chun Hsieh

**Affiliations:** Department of Electrical Engineering, National Chin-Yi University of Technology, Taichung 41170, Taiwan; E-Mails: zi_xiang2008@hotmail.com (T.-H.H.); rxz3145@yahoo.com.tw (C.-C.H.)

**Keywords:** chaotic system, Bluetooth, communications security, Particle Swarm Optimization algorithm, PID controller

## Abstract

This study used the complex dynamic characteristics of chaotic systems and Bluetooth to explore the topic of wireless chaotic communication secrecy and develop a communication security system. The PID controller for chaos synchronization control was applied, and the optimum parameters of this PID controller were obtained using a Particle Swarm Optimization (PSO) algorithm. Bluetooth was used to realize wireless transmissions, and a chaotic wireless communication security system was developed in the design concept of a chaotic communication security system. The experimental results show that this scheme can be used successfully in image encryption.

## 1. Introduction

The chaos phenomenon was first proposed by Lorenz using the simulation equation of the atmosphere, but it did not attract much attention from scientists until Feigenbaum proposed the general theory of chaos phenomenon. Chaos is a phenomenon that seems disorderly but contains rules. It is a complex dynamic non-periodic and nonlinear system that cannot be explained by single data, and should be analyzed using overall continuous data. It has a very extensive Fourier spectrum, and has a fractal in the phase plane. The key in its state response is the initial value of the system. If a system has different initial values, the response varies largely; this phenomenon is called the butterfly effect [1–3].

At present, many scientists have applied the chaotic system concept to image encryption. This work can be briefly introduced as follows: Gao *et al.* proposed an image encryption algorithm, which randomly shuffles the matrices of image pixel positions, and uses a hyper-chaotic system to mix the relation between plainimage and cipherimage [4]. Guan *et al.* used a 2D cat map in random image pixel positions, and the output pixels of discrete Chen's system to cover the original pixel value [5]. Lian proposed an image encryption algorithm based on a spatiotemporal chaos system. The spatiotemporal lattices were used to generate a random sequence, and this sequence was used to select cryptographic parameters in each segment [6] Pareek *et al.* proposed an image encryption method based on chaotic Logistic maps, using one 80-bit external key and two chaotic Logistic maps. The initial conditions of the Logistic maps were obtained using the external key, and eight different operation types were used for the encryption of images [7]. Chen *et al.* proposed a real-time secure image encryption algorithm extending the image encryption algorithm for two-dimensional chaotic maps to three-dimensional ones. This method used a three-dimensional cat map in random image pixel positions, and employed the relation between another chaotic map encryption and the original image for confusion [8]. Although the above methods are all feasible, they are too complex and have high commercialization costs. This study used a simple method with the chaotic system to encrypt and decrypt images.

The chaotic synchronization system generally consists of a master chaotic system, a slave chaotic system, and a controller synchronizing the master and slave systems. The controller processes the signals of the master chaotic system, and transmits them to the slave chaotic system, so as to synchronize the trajectories of the two systems [9–13]. In this study, a PID controller was used to control the two systems, and the three parameters *K_p_, K_i_* and *K_d_* of the PID were selected using a Particle Swarm Optimization (PSO) algorithm. The optimum parameters were thus obtained. Finally, the LabVIEW software was used to integrate the cryptological concept with the chaotic synchronization system into a chaotic synchronous cryptographic system, which was applied to the wireless communication secrecy for image encryption.

## 2. System Description and Formulation Problem

In order to observe the procedure of chaotic synchronization, the Master/Slave system of a single input single output (SISO) is used. The differential equations [14,15] are described below:

Master System:

(1) $$\{\begin{array}{l} {\dot{x}}_{m}(t)=f(t,x_{m})\\ y_{m}(t)=Cx_{m} \end{array}$$

Slave System:

(2) $$\{\begin{array}{l} {\dot{x}}_{s}(t)=f(t,x_{s})+Bu(t)\\ y_{s}(t)=Cx_{s} \end{array}$$

Among which, *x_m_(t)*=[*x_m1_,x_m2_,x_m3_*]∈*R^n^* and *x_s_(t)*=[*x_s1_,x_s2_,x_s3_*]∈*R^n^* are the status values of Master System and Slave System, *f:R*×*R^n^*→*R^n^* is the nonlinear function, *y_m_(t)*∈*R* and *y_s_(t)*∈*R* are the outputs of Master System and Slave System, B∈R^n×1^ and C∈R^1×n^, *u*∈R is the controller in the Slave system, the control objective is:

(3) $$\underset{t\to \infty }{\text{lim}}\left\|x_{m}(t)-x_{s}(t)\right\|\to 0$$

Since the initial value conditions of the master system and slave system are different, the synchronous controller is added, and the slave system is driven by the signals of the synchronous controller. Thus, the master system and the slave system have coincident response, that is synchronization. The state error of master and slave systems is defined as follows:

(4) $$e_{1}=x_{m,1}-x_{s,1},e_{2}=x_{m,2}-x_{s,2},\cdots ,e_{n}=x_{m,n}-x_{s,n}$$

The primary objective of this system is to propose a simple and effective PID controller, using a PSO algorithm to obtain the optimum PID parameter values to synchronize two identical chaotic systems with different initial conditions. The *u* in Equation (2) is the PID controller ensuring the synchronization effect based on the PSO algorithm. In order to determine the u of PID controller, the output error signal *y_e_* = *y_m_* − *y_s_* is defined first, and the PID controller and input *y_e_(t)* and output *u(t)* can be expressed in continuous form as the following equation:

(5) $$u(t)=K_{p}\left[y_{e}(t)+\frac{1}{T_{i}}\int_{0}^{t}y_{e}(t)dt+T_{d}\frac{d}{dt}y_{e}(t)\right]$$

where *K_p_* is the proportional gain, *T_i_* is the constant of integral time, and *T_d_* is the constant of derivative time.

As the PID controller is realized in digital control, the continuous PID controller is converted into a discrete PID. Equation (5) can be changed to the following form [16]:

(6) $$u(k)=K_{p}\left[y_{e}(k)+\frac{1}{T_{i}}S(k)+\frac{T_{d}}{T}[y_{e}(k)-y_{e}(k-1)]\right]$$

where *u(k)* is the output of controller from k samples, *S(k)* is the sum of the deviations, *T* is the sampling time. Equation (6) can be expressed as follows:

(7) $$u(k)=K_{p}y_{e}(k)+K_{i}S(k)+K_{d}[y_{e}(k)-y_{e}(k-1)]$$

where $K_{i}=K_{p}\frac{1}{T_{i}}$ is the integral gain, and $K_{d}=K_{p}\frac{T_{d}}{T}$ is the derivative gain.

In general cases, the adjustment of PID controller involves selecting proper parameters *K_p_, K_i_, K_d_* to ensure the system has better control performance, and the performance standard (objective function) can be defined according to the required specifications. There are two performance indexes: Integrated Squared Error (ISE) and Integrated Absolute Error (IAE). Their mathematical definitions are shown below:

(8) $$\text{ISE}=\int_{0}^{\infty }e^{2}(\tau )d\tau$$

(9) $$\text{IAE}=\int_{0}^{\infty }\left|e(\tau )\right|d\tau$$

This paper uses IAE as the objective function (OF), so Equation (9) is changed to the following equation:

(10) $$OF=\text{IAE}=\int_{0}^{\infty }\left|\left\|E(\tau )\right\|\right|d\tau$$

According to PSO algorithm, an ideal gain parameter adjustment method for PID controller is determined to minimize the objective function.

## 3. Solve Optimization Problem Using PSO Algorithm

**Figure 1.**
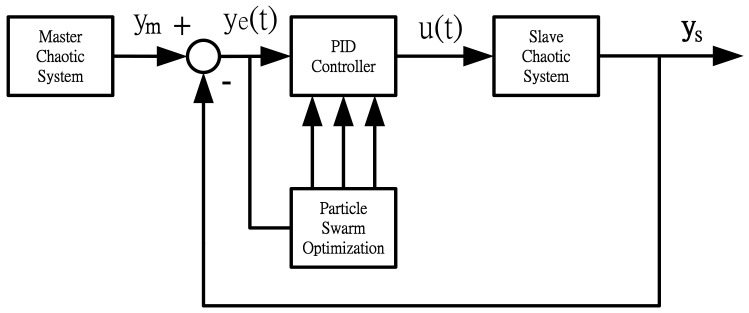
Block diagram of PID controlled chaotic synchronization system of the PSO algorithm.

As the PSO algorithm has memory and distributed search features [17–19], it has high accuracy in the optimization of complicated systems, so the PSO algorithm is used in our system to solve the parametric problem of the PID controller. The PID control system consists of a master chaotic system, a slave chaotic system, a PID controller, and the PSO algorithm. The corresponding block diagram is shown in Figure 1.

In Figure 1, *y_m_* is the output of master chaotic system, *y_s_* is the output of slave chaotic system, *y(e)* is the output error between the master chaotic system and the slave chaotic system, *u(t)* is the control output of PID controller defined as Equation (7). The optimum parameters *K_p_, K_i_, K_d_* of PID controller are obtained using the PSO algorithm to search for the convergent minimum value of performance index of IAE defined as Equation (10).

## 4. Optimization Problem Formulation and Procedure

The PSO algorithm is used to solve the parametric optimization as mentioned in the previous section. First, *K∈S* is defined, let *K* be continuous differentiable matrix value function, *S* = *{z*≤*R^3^*∣*0*≤*z_i_*≤*z_max_,z_max_*<∞*, i* = *1,2,3}, z_max_* is the search area. The result of optimization problem includes z*=[K_p_*,K_i_*,K_d_*]*∈S*, such a parameter value can minimize IAE. This optimization problem is described as mathematical expression for accuracy, namely, to determine a parameter z*∈S to minimize IAE:

(11) $$\begin{array}{ccc} OF=\text{IAE}=\int_{0}^{\infty }\left|\left\|E(\tau )\right\|\right|d\tau & , & z^{*}\in s \end{array}$$

**Figure 2.**
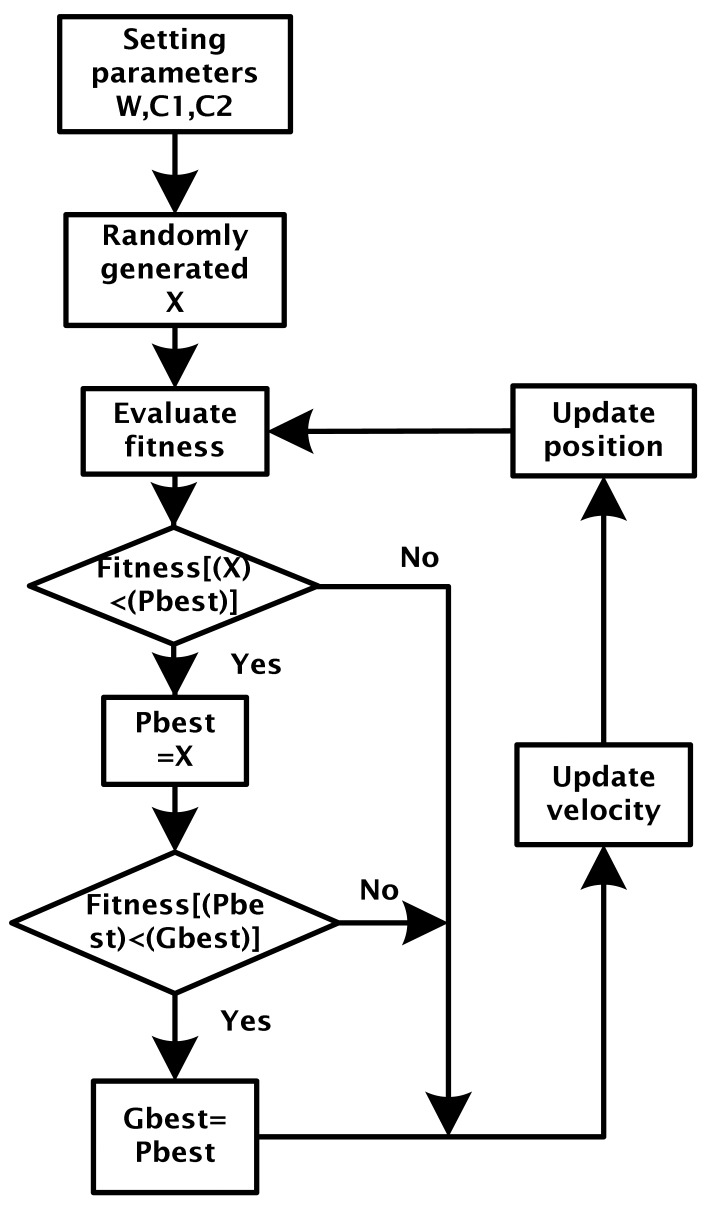
PSO algorithm process block diagram.

According to “PSO in Electromagnetics” [20], a block flow diagram can be induced from the PSO algorithm, as shown in Figure 2.

The velocity update equation is shown below

(12) $$V_{i}=W\times V_{i}+C_{1}\times R_{\text{and}}\times (P_{\text{best}}-X_{i})+C_{2}\times R_{\text{and}}\times (G_{\text{best}}-X_{i})$$

where:

*V_i_*: Velocity of each particle
*i*: Number of particle
W: Inertia Weight
*C*_1_, *C*_2_: Learning constant
*R_and_*: Random number between 0 and 1
*P_best_*: The optimum position of each particle up to now
*G_best_*: The optimum position of all particles up to now
*X_i_*: The present position of each particle

Position update equation of each particle point in particle swarm:

(13) $$X_{i}=X_{i}+V_{i}$$

## 5. Image Encryption and Decryption

This study used the Sprott chaotic synchronization system and cryptology concept to design a wireless communication secrecy system, and employed LabVIEW software to transmit images in wireless mode. The data were encrypted and decrypted by computer to computer. Bluetooth, which is a wireless personal LAN, was used for wireless transmission. The transmission frequency of Bluetooth was 2.45 GHz. Besides digital data transmission, sound transmission was also available. The transmission speed of Bluetooth was 2∼3 Mb per second, and encryption protection could be set. The frequency changed 1,600 times per min, so it was unlikely to be intercepted and was free from interference from electromagnetic waves. Each Bluetooth-based connecting device had a 48-bit address according to the IEEE 802 standard. It could connect one or many devices, and the maximum transmission range was about 100 m. This study used a D401 mini-Bluetooth receiver V2.0 EDR, with a transmission distance of 20 m, and a transmission speed of 2.1 Mb per second.

**Figure 3.**
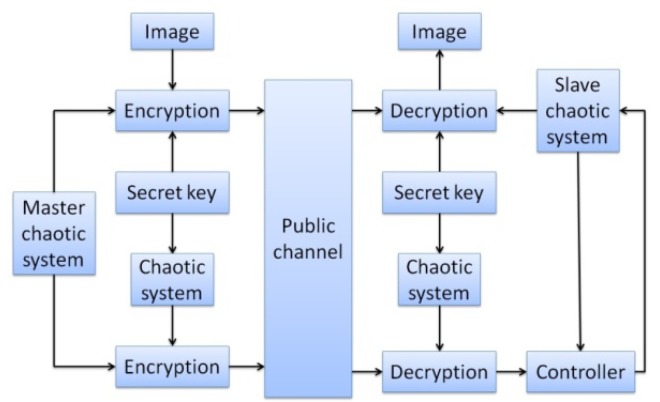
Structure diagram of Image encryption and decryption of chaotic synchronous cryptographic system.

**Figure 4.**
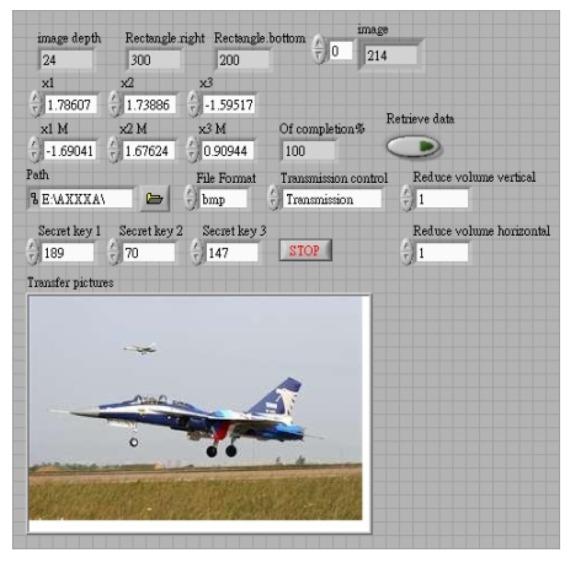
Transmission interface.

**Figure 5.**
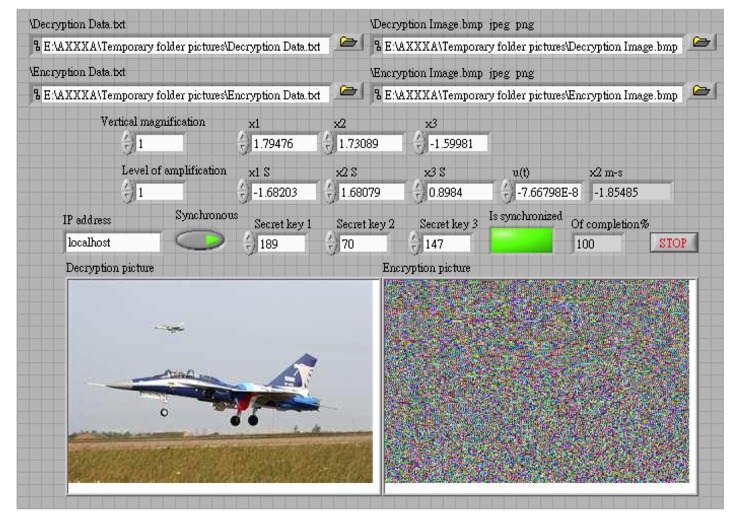
Reception control interface.

In the image encryption and decryption, the user has to enter a key, and this value is mixed with the chaotic signal. A password is then generated randomly as the chaotic signal changes, and is mixed with the pixels of the original image. The chaotic signal is used to select 16 different data ordering modes. The RGB values of pixels are combined by staggered arrangement for encryption and decryption. The initial value of this chaotic system is generated randomly. In synchronous signal transmission, another chaotic system is used for encryption and decryption, and the initial value of this chaotic signal is obtained from the key entered by the user. The system structure is shown in Figure 3, and the control interface of system is shown in Figures 4 and 5.

## 6. Simulation and Experimental Results

This study used LabVIEW to design the PID controller, applied a PSO algorithm to obtain the optimum parameter values and perform synchronization control for the Sprott [13,14] chaotic circuit system. The system equations are shown below:

Master:

(14) $$\begin{array}{l} {\dot{x}}_{m,1}=x_{m,2}\\ {\dot{x}}_{m,2}=x_{m,3}\\ {\dot{x}}_{m,3}=-1.2x_{m,1}-x_{m,2}-0.6x_{m,3}+2\cdot \text{sign}\left(x_{m,1}\right) \end{array}$$

**Figure 6.**
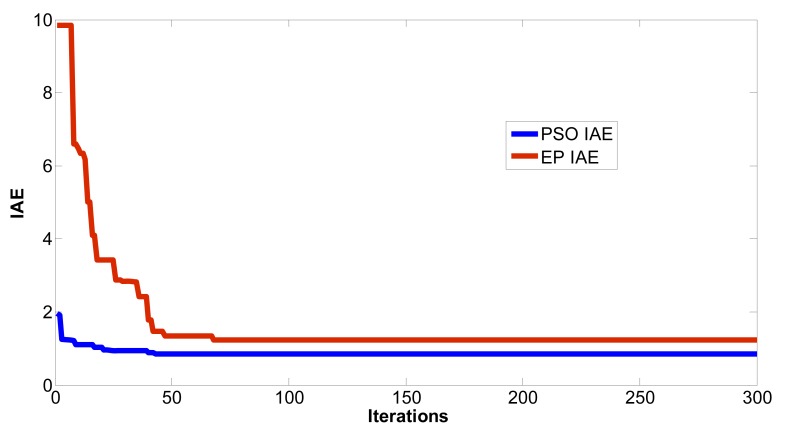
IAE convergence curve.

**Figure 7.**
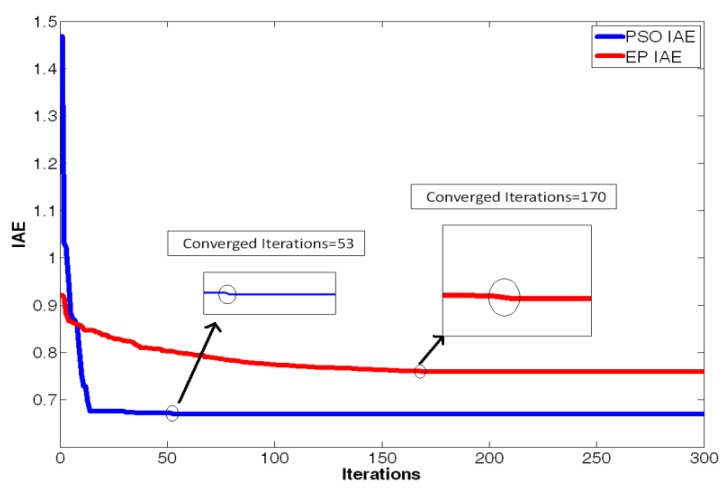
IAE convergence curve by PSO and EP with the initial conditions [xm1(0), xm2(0), xm3(0)] = [0.1, 0.1, 0.1] and [xs1(0), xs2(0), xs3(0)] = [−1, −1, −1].

**Figure 8.**
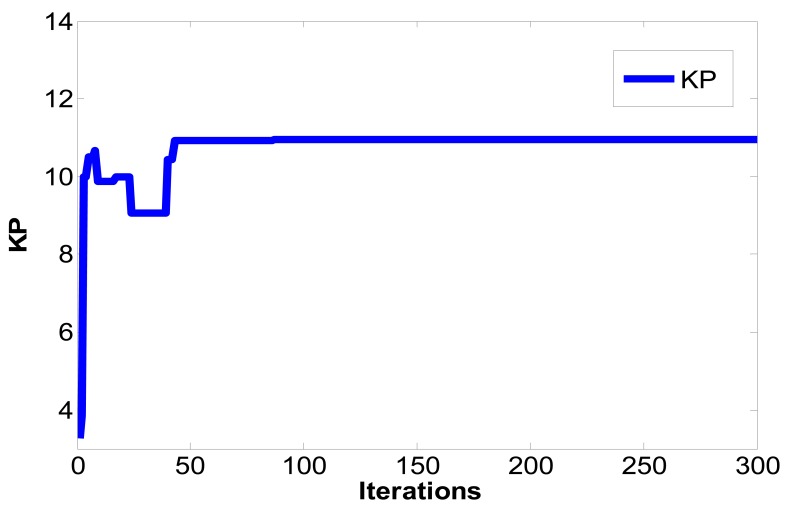
*k_p_* convergence curve.

**Figure 9.**
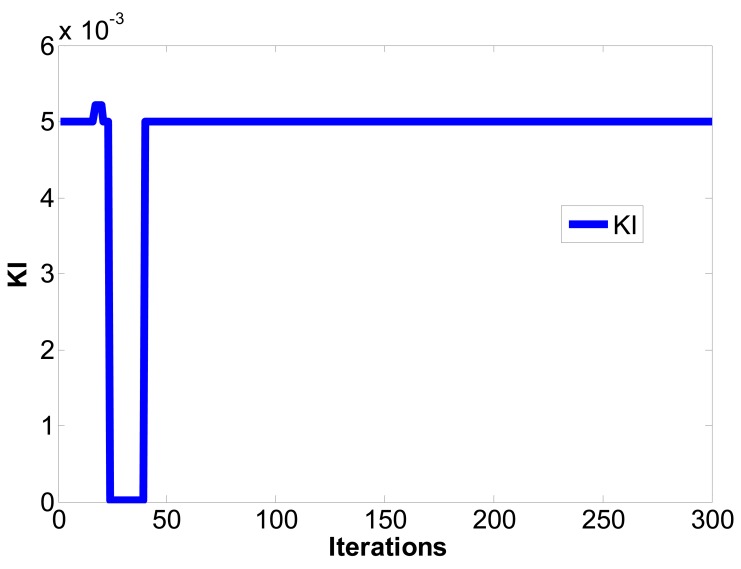
*k_i_* convergence curve.

**Figure 10.**
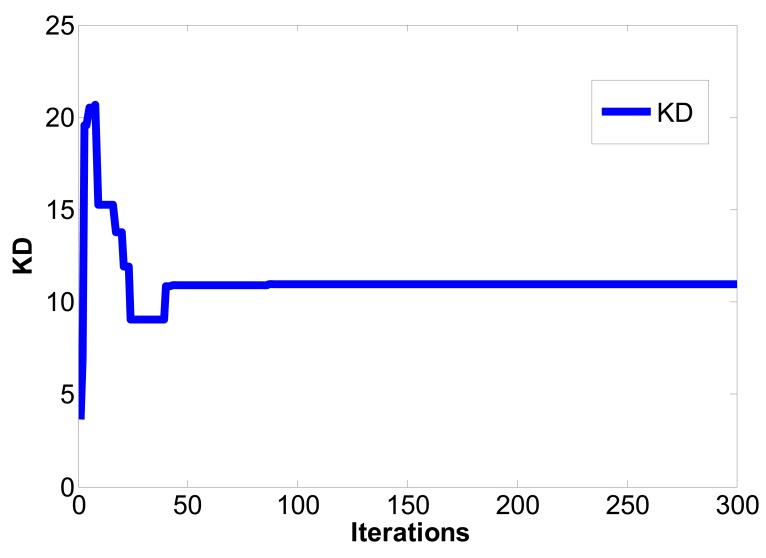
*k_d_* convergence curve.

**Figure 11.**
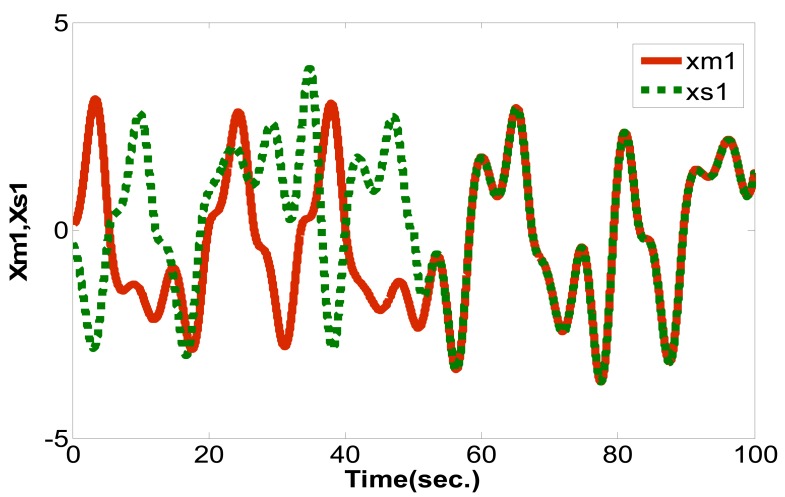
*x_m1_* and *x_s1_* synchronization curve.

**Figure 12.**
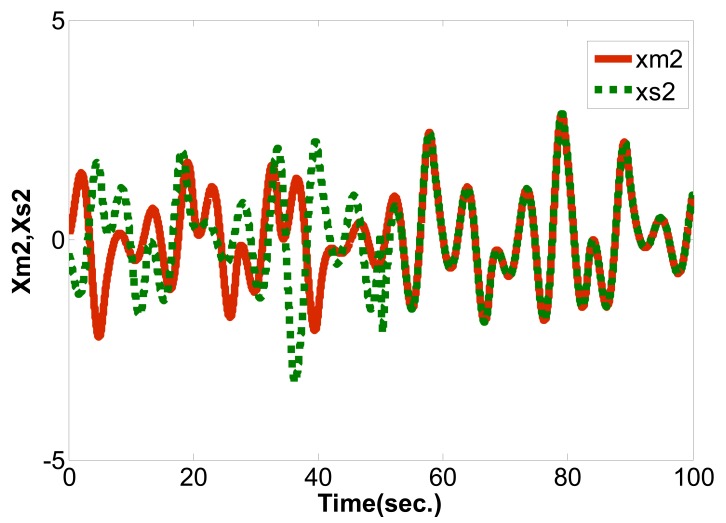
*x_m2_* and *x_s2_* synchronization curve.

**Figure 13.**
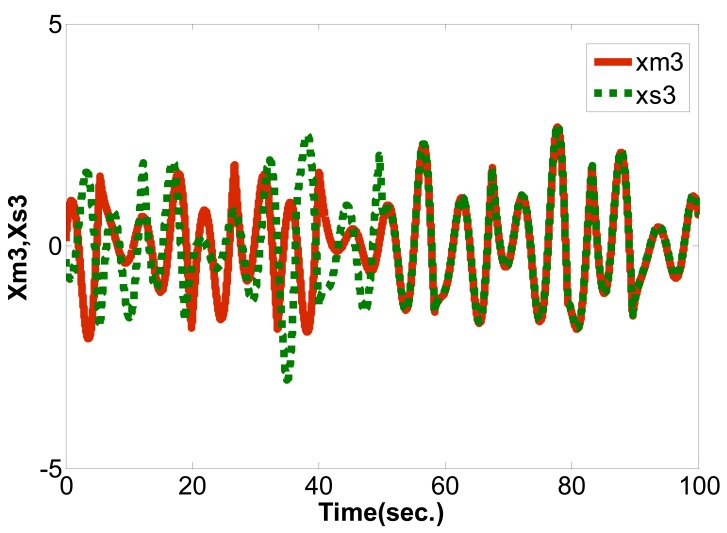
*x_m3_* and *x_s3_* synchronization curve.

Slave:

(15) $$\begin{array}{c} {\dot{x}}_{s,1}=x_{s,2}\\ {\dot{x}}_{s,2}=x_{s,3}+u(t)\\ {\dot{x}}_{s,3}=-1.2x_{s,1}-x_{s,2}-0.6x_{s,3}+2\cdot \text{sign}\left(x_{s,1}\right) \end{array}$$

where the *x_m_, x_s_* derived from each *ẋ_m_, ẋ_s_* are related to Time *t*, and *u(t)* is the controller added. The Master and Slave trajectories demonstrate chaotic motions when the controller *u(t)* = 0, the control objective is:

(16) $$\underset{t\to \infty }{\lim}\left\|x_{m}(t)-x_{s}(t)\right\|\to 0$$

where the master chaotic system and slave chaotic system come to synchronization. MATLAB and Simulink were used for simulation. If the initial conditions of the master chaotic system and slave chaotic system are [*x_m1_(0),x_m2_(0),x_m3_(0)*] = [0.1 0.1 0.1] and [*x_s1_(0),x_s2_(0),x_s3_(0)*] = [−0.5 −0.5 −0.5], and the particle population and number of iterations of optimization problem are set as 10 and 300 to determine the optimum parameter values of the PID controller. The IAE converges at 45 iterations as calculated by the PSO algorithm and 68 iterations as calculated by the EP algorithm. The steady state values of IAE are 0.8451 by PSO and 1.231 by EP, as shown in Figure 6. Compared with [16] and [21] under the same initial conditions, it can be seen that the IAE convergence speed of the PSO algorithm is faster than the evolutionary programming (EP) algorithm, as shown in Tables 1, 2 and Figure 7. Therefore, we can find that the PSO algorithm calculation performance is better than the EP algorithm in this study. The *k_p_,k_i_,k_d_* parameter values of PID controller by PSO algorithm are z*=[K_p_*,K_i_*,K_d_*]=[10.9554 0.005 10.9504], as shown in Figures 8–10. The simulation results of adding synchronizing signal in after 50 s of Sprott are shown in Figures 11–13.

**Figure 14.**
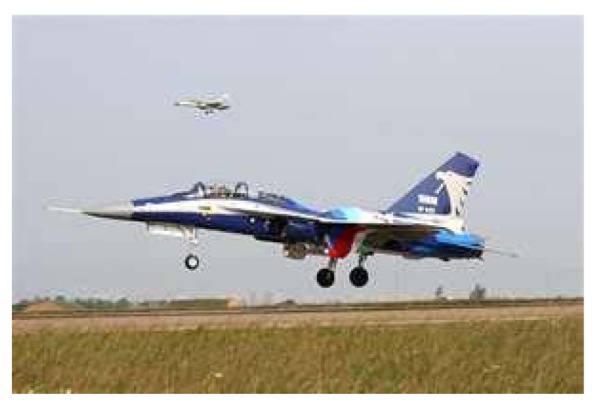
Original picture of an airplane.

**Figure 15.**
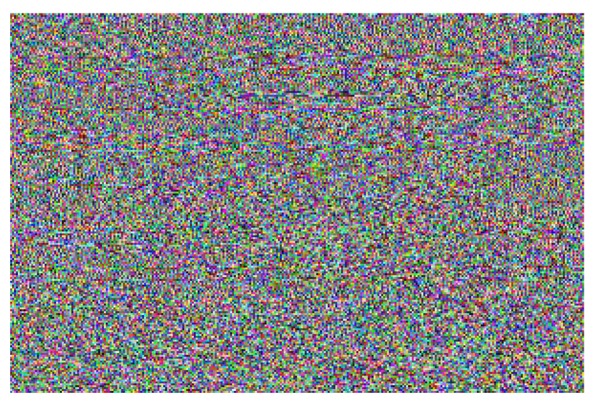
Post-encryption effect.

**Figure 16.**
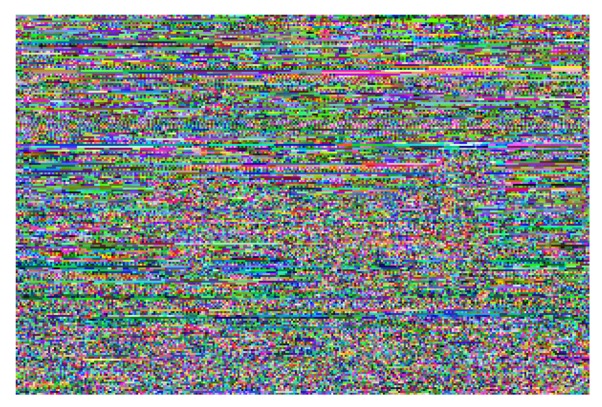
Decryption effect when the key entered is incorrect.

**Figure 17.**
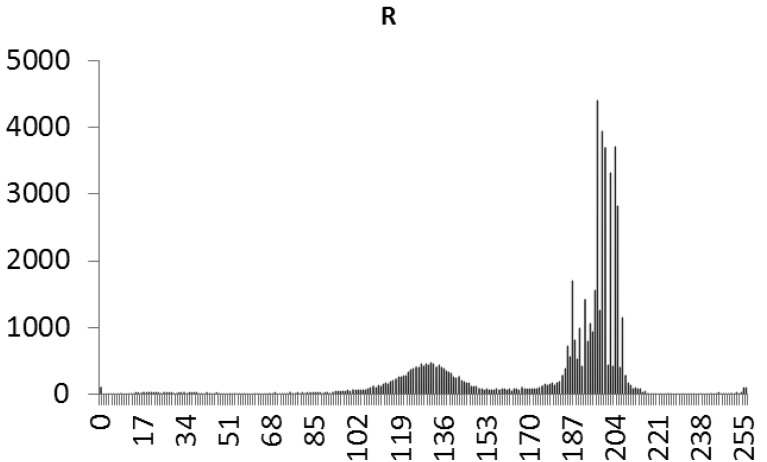
Statistical chart of R value distribution of the original image.

**Figure 18.**
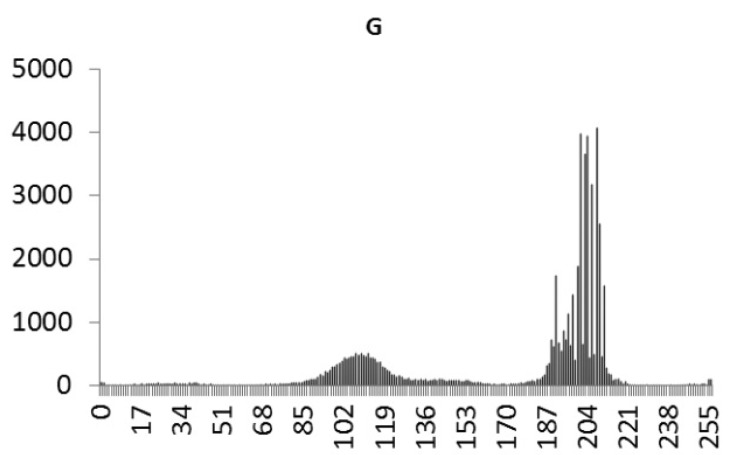
Statistical chart of G value distribution of the original image.

**Figure 19.**
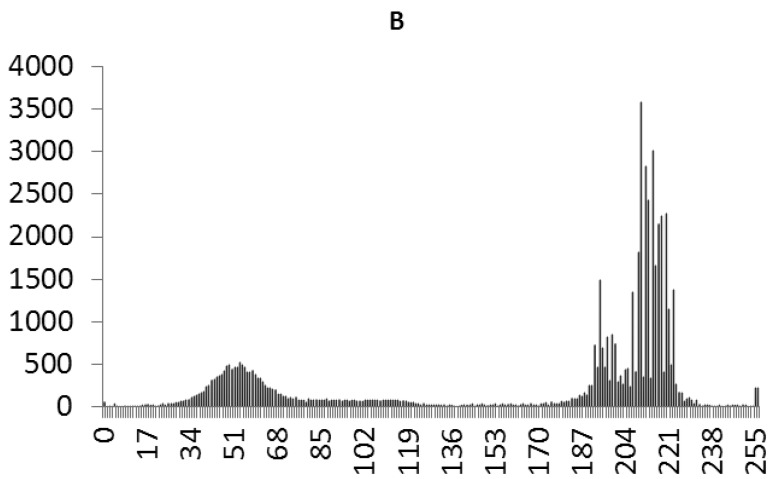
Statistical chart of B value distribution of the original image.

**Figure 20.**
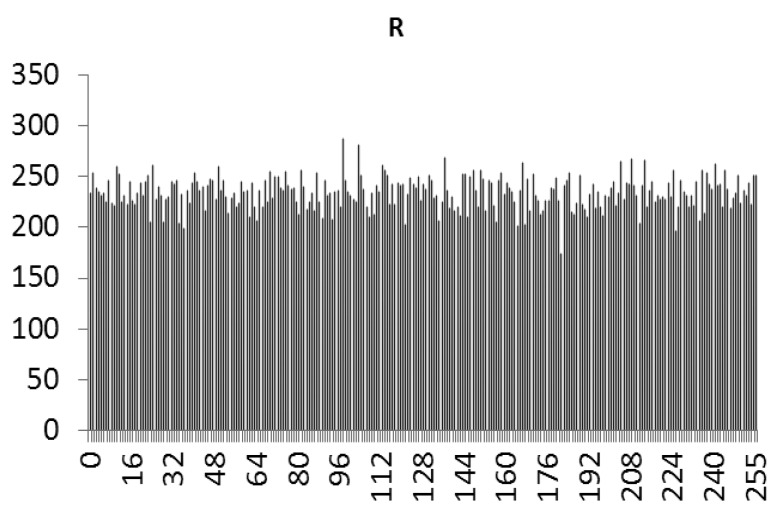
Statistical chart of R value distribution after encryption.

**Figure 21.**
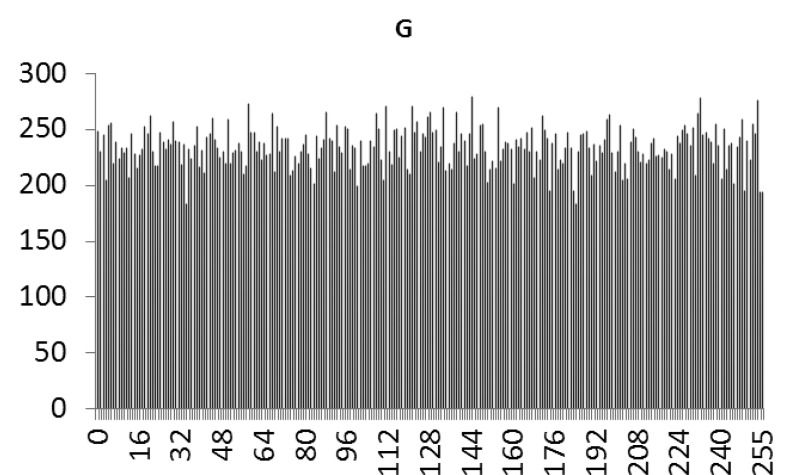
Statistical chart of G value distribution after encryption.

**Figure 22.**
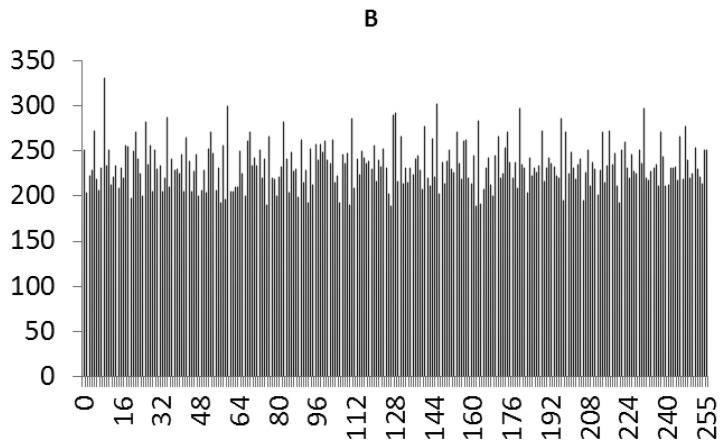
Statistical chart of B value distribution after encryption.

**Figure 23.**
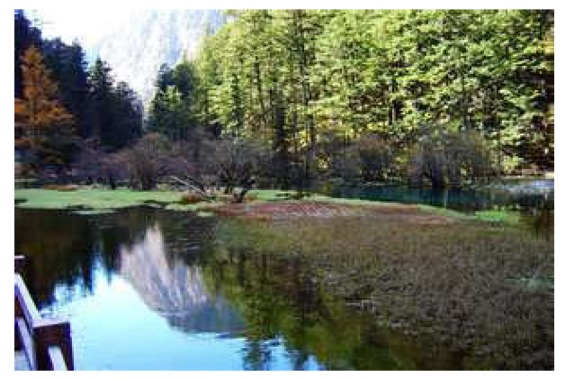
Original scenery picture.

**Figure 24.**
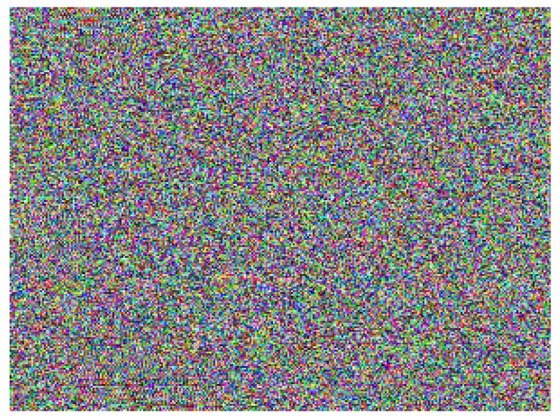
Post-encryption effect.

**Figure 25.**
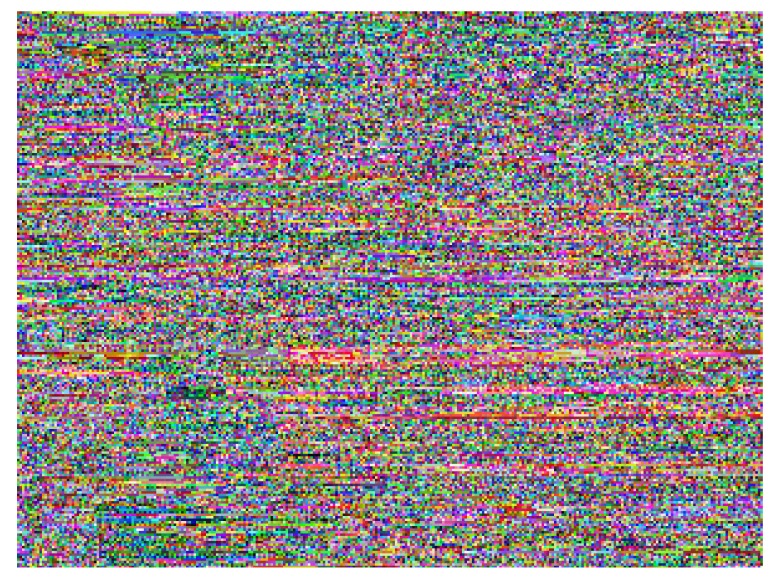
Decryption effect when the key entered is incorrect.

**Figure 26.**
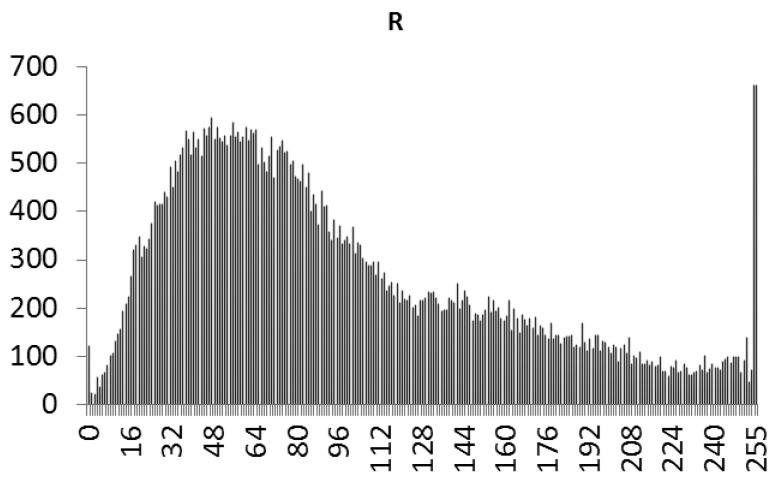
Statistical chart of R value distribution of the original image.

**Figure 27.**
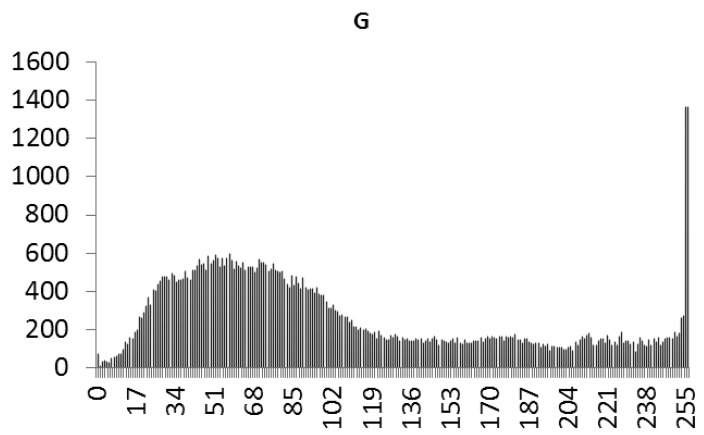
Statistical chart of G value distribution of the original image.

**Figure 28.**
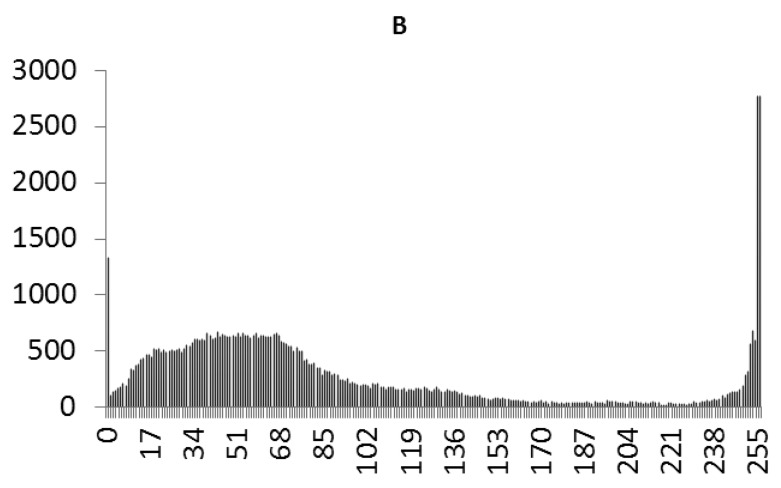
Statistical chart of B value distribution of the original image.

**Figure 29.**
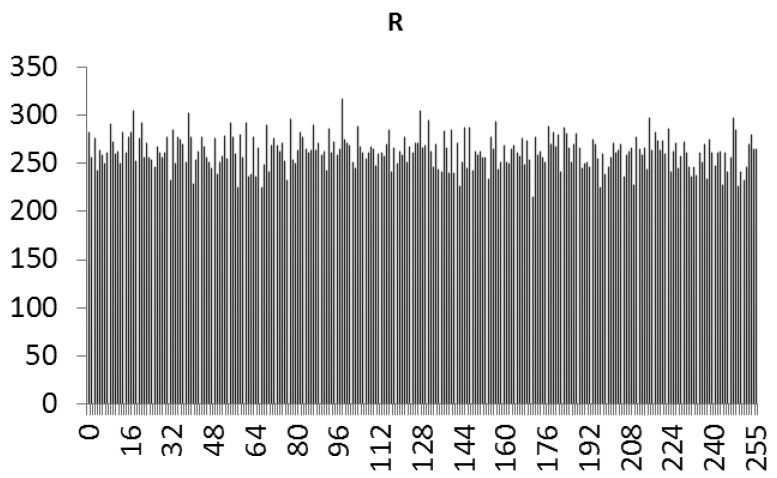
Statistical chart of R value distribution after encryption.

**Figure 30.**
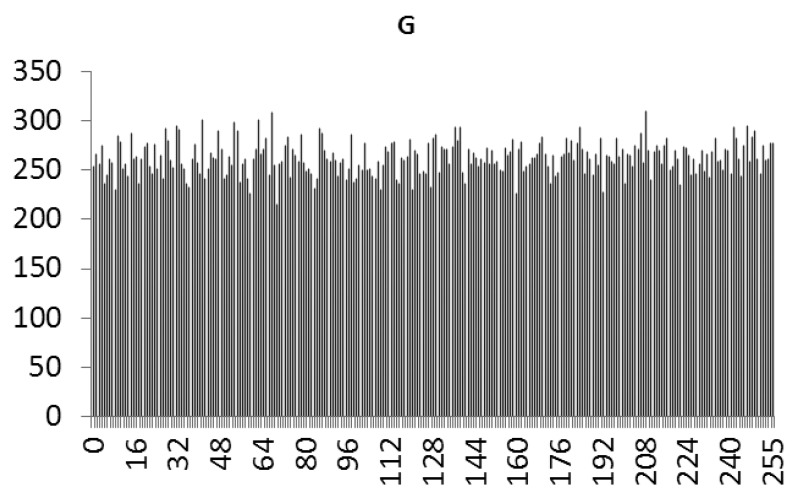
Statistical chart of G value distribution after encryption.

**Figure 31.**
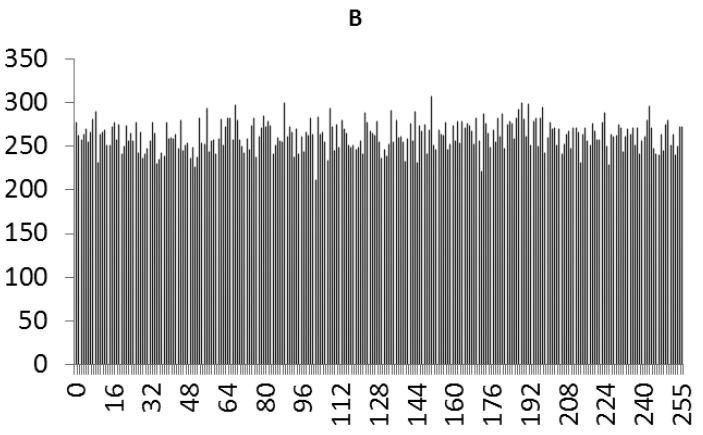
Statistical chart of B value distribution after encryption.

This study produced two images for encryption and decryption. One was a picture of an airplane, and the experimental results are shown in Figures 14–22. Figure 14 is the original airplane picture to be transmitted, and the image is disorganized into Figure 15 after encryption processing. Figure 16 shows the decryption when the key entered is incorrect. Figures 17–19 show the RGB distribution of the original picture. Figures 20–22 show the RGB distribution of the encrypted picture. Another image is a scenery picture, and the experimental results are shown in Figures 23–25. Figure 23 is the original scenery picture to be transmitted, and the image is disorganized into Figure 24 after encryption processing. Figure 25 shows the decryption when the key entered is incorrect. Figures 26–28 show the RGB distribution of the original picture. Figures 29–31 show the RGB distribution of the decrypted picture.

## 7. Conclusions

This study successfully used PSO to obtain the optimum parameter values of a chaotic synchronization PID controller, and applied it in chaotic communication secrecy. A traditional PID controller can only be used in fixed systems, and must be redesigned if it is to be used in different systems, which consumes a high hardware setting time and cost. This study used LabVIEW, instead of the traditional PID controller, so that when it is applied in other systems, only the parameters of the PID controller need to be changed, so the time and cost can be reduced. Bluetooth was used to realize wireless transmissions. In the application of wireless communications, future studies can focus on the encryption and decryption of images in order to improve the security of wireless transmission.

## Figures and Tables

**Table 1.** The convergence situation of PSO *vs*. EP with the initial conditions [xm1(0), xm2(0), xm3(0)] = [0.1, 0.1, 0.1] and [xs1(0), xs2(0), xs3(0)] = [−1, −1, −1].

	**Converged iterations**	**Steady state of IAE**
**The EP method in reference** [16]	170	0.7592
**The PSO method in this paper**	53	0.6697

**Table 2.** The convergence situation of PSO *vs*. EP with the initial conditions [xm1(0), xm2(0), xm3(0)] = [0.1, 0.1, 0.1] and [xs1(0), xs2(0), xs3(0)] = [−1, −2, 1].

	**Converged iterations**	**Steady state of IAE**
**The EP method in reference** [21]	80	0.6726
**The PSO method in this paper**	26	0.4132

## References

[1] Lerescu A.I., Constandache N., Oancea S., Grosu I. (2004). Collection of master–slave synchronized chaotic systems. Chaos Solitons Fractals.

[2] Chua L.O., Lin G.N. (1990). Canonical realization of Chua's circuit family. IEEE Trans. Circuits Syst..

[3] Lorenz E.N. (1963). Deterministic non-periodic flow. J. Atmos. Sci..

[4] Gao T., Chen Z. (2008). A new image encryption algorithm based on hyper-chaos. Phys. Lett. A.

[5] Guan Z.-H., Huang F., Guan W. (2005). Chaos based image encryption algorithm. Phys. Lett. A.

[6] Lian S. (2009). Efficient image or video encryption based on spatiotemporal chaos system. Chaos Solitons Fractals.

[7] Pareek N.K., Patidar V., Sud K.K. (2006). Image encryption using chaotic logistic map. Image Vis. Comput..

[8] Chen G., Mao Y., Chui C.K. (2004). A symmetric image encryption scheme based on 3D chaotic cat maps. Chaos Solitions Fractals.

[9] Chen S., Lü J. (2002). Synchronization of an uncertain unified chaotic system via adaptive control. Chaos Solitons Fractals.

[10] Zhang H., Ma X.K., Liu W.Z. (2004). Synchronization of chaotic systems with parametric uncertainty using active sliding mode control. Chaos Solitons Fractals.

[11] Wen G., Wang Q.G., Lin C., Han X., Li G. (2006). Synthesis for robust synchronization of chaotic systems under output feedback control with multiple random delays. Chaos Solitons Fractals.

[12] Pecora L.M., Carroll T.L. (1990). Synchronization in chaotic systems. Phys. Rev. Lett..

[13] Ott E., Grebogi C., Yorke J.A. (1990). Controlling chaos. Phys. Rev. Lett..

[14] Sprott J.C. (2000). A new class of chaotic circuits. Phys. Lett. A.

[15] Almeida D.I.R., Alvarez J., Barajas J.G. (2006). Robust synchronization of Sprott circuits using sliding mode control. Chaos Solitons Fractals.

[16] Yau H.T., Pu Y.C., Li S.C. (2011). An FPGA-based PID controller design for chaos synchronization by evolutionary programming. Discret. Dyn. Nat. Soc..

[17] Chang J.C. (2012). DOA Estimation for local scattered cdma signals by particle swarm optimization. Sensors.

[18] Zhang Y., Wu L. (2011). Crop classification by forward neural network with adaptive chaotic particle swarm optimization. Sensors.

[19] Wang X., Wang S., Ma J.J. (2007). An improved co-evolutionary particle swarm optimization for wireless sensor networks with dynamic deployment. Sensors.

[20] Robinson J., Rahmat-Samii Y. (2004). Particle swarm optimization in electromagnetics. IEEE Trans. Antennas Propag..

[21] Chen H.C., Chang J.F., Yan J.J., Liao T.L. (2008). EP-based PID control design for chaotic synchronization with application in secure communication. Expert Syst. Appl..

